# Modulation of Ethylene and Ascorbic Acid on Reactive Oxygen Species Scavenging in Plant Salt Response

**DOI:** 10.3389/fpls.2019.00319

**Published:** 2019-03-18

**Authors:** Juan Wang, Rongfeng Huang

**Affiliations:** ^1^Biotechnology Research Institute, Chinese Academy of Agricultural Sciences, Beijing, China; ^2^National Key Facility for Crop Gene Resources and Genetic Improvement, Beijing, China

**Keywords:** ethylene, AsA, salt stress, ROS scavenging, homeostasis

## Abstract

Salt stress causes retarded plant growth and reduced crop yield. A complicated regulation network to response to salt stress has been evolved in plants under high salinity conditions. Ethylene is one of the most important phytohormones, playing a major role in salt stress response. An increasing number of studies have demonstrated that ethylene modulates salt tolerance through reactive oxygen species (ROS) homeostasis. Ascorbic acid (AsA) is a non-enzymatic antioxidant, contributing to ROS-scavenging and salt tolerance. Here, we mainly focus on the advances in understanding the modulation of ethylene and AsA on ROS-scavenging under salinity stress. We also review the regulators involved in the ethylene signaling pathway and AsA biosynthesis that respond to salt stress. Moreover, the AsA pool is affected by many environmental conditions, and the potential role of ethylene in AsA production is also extensively discussed. Novel insights into the roles and mechanisms of ethylene in AsA-mediated ROS homeostasis will provide critical information for improving crop salt tolerance.

## Introduction

According to the report from the Food and Agricultural Organization of the United Nations, there will be challenges related to the productivity of crops to supply more food for an additional 2.3 billion people by 2050. Crop yield is greatly affected by various abiotic stresses like drought, high salinity, cold, and heat (Zhu, 2016). For instance, high salinity stress disturbs plant physiological processes through osmotic stress and ionic toxicity, causing reductions in both crop growth and yield (Yang and Guo, 2018a,b). For better utilization of salt-affected lands, it is of great help to develop crops with improved salt tolerance through molecular-assistant breeding to reveal the underlying mechanisms involved in plant response to salt stress.

Salt stress responses generally correlate with the regulations of phytohormones, including abscisic acid (ABA), jasmonic acid, gibberellin, and ethylene (Julkowska and Testerink, 2015). Although there exists opposite modulation between monocot and dicot plants (Peng et al., 2014; Yang et al., 2015), increasing investigations have revealed that ethylene-conferred salt tolerance is mediated by deterring reactive oxygen species (ROS) homeostasis (Jiang et al., 2013; Li et al., 2014; Peng et al., 2014; Yang and Guo, 2018b). Under salt stress, ROS, including hydrogen peroxide, superoxide anions and hydroxyl radicals, accumulate and damage cellular structure (Ahanger et al., 2017). ROS plays a dual role in response to stresses as toxic by products and major signal (Qi et al., 2018), and the excess ROS could be scavenged through enzymatic and non-enzymatic antioxidant defense systems (You and Chan, 2015). Accumulating investigations have revealed that ascorbic acid (AsA) is an essential compound of non-enzymatic antioxidant in plants, functioning in plant growth, hormone signaling, and stress response (Bulley and Laing, 2016; Mellidou and Kanellis, 2017; Vidal-Meireles et al., 2017). AsA plays especially critical roles in the fine control of ROS homeostasis to improve salt tolerance (Smirnoff, 2000; Shalata and Neumann, 2001; Zhang et al., 2012; Wang J. et al., 2013), implying that AsA has an essential modulation in salt response. Considering there are some reviews about ethylene-modulated salt response (Zhang M. et al., 2016), this mini review will focus on the advances in understanding the modulation of ethylene on AsA biosynthesis and ROS-scavenging under salinity stress.

### The Regulation of Ethylene on ROS Homeostasis Is Tightly Associated With the Plant Response to Salt Stress

Gaseous phytohormone ethylene plays an important role in mediating numerous specific growth and development processes (Wang et al., 2018), especially in response to various stress conditions (Wang F. et al., 2013; Dubois et al., 2018). The biosynthesis and signaling pathway of ethylene have been well established (Guo and Ecker, 2004). After recognition of ethylene by endoplasmic reticulum membrane-associated receptors, the interaction of ethylene receptors with CONSTITUTIVE TRIPLE RESPONSE1 (CTR1) will be released, and the phosphorylation of CTR1 on ETHYLENE INSENSITIVE 2 (EIN2) will be liberated. Then, the C-terminal of EIN2 is generated by an unknown mechanism and is transported to cytoplasmic processing-body (P-body) to repress translation of EIN3 BINDING F-BOX1/2 (EBF1/2), which mediates the proteasomal degradation of EIN3 and EIN3-LIKE 1 (EIL1), resulting in the stability of EIN3/EIL1 proteins and promotion of ethylene response (Li et al., 2015). APETALA2/ETHYLENE RESPONSE FACTORS (AP2/ERFs) are one of the most important transcription factor families, regulating multiple developmental and stress response processes (Phukan et al., 2017), most of which are downstream targets of ethylene signaling (Liu et al., 2016).

Ethylene has long been known for modulating salt stress response (Cao et al., 2007). For instance, blocked ethylene signaling confers reduced salt tolerance to *Arabidopsis* (Achard et al., 2006; Peng et al., 2014). *ein3 eil1* double mutants and other ethylene signaling-related mutants showed enhanced sensitivity to salt stress. In contrast to this modulation, ethylene displays a negative role in rice (Yang et al., 2015). *OsEIL1* and *OsEIL2* RNAi transgenic plants displayed increased salt tolerance. The regulation of ethylene biosynthesis also plays different roles in salt tolerance between *Arabidopsis* and rice (Jiang et al., 2013; Li et al., 2014). Ethylene Overproducer 1 (ETO1) plays a positive role in salt response through promoting ROS generation, followed with Na^+^/K^+^ homeostasis modulation in *Arabidopsis*. However, SALT INTOLERANCE 1 (SIT1) negatively regulates salt response due to activation on MITOGEN-ACTIVATED PROTEIN KINASE 3/6 (MPK3/6) in rice, which promotes ethylene and ROS overproduction (Table 1). Thus, the different mechanisms of ethylene-directed salt response between monocot and dicot plants remain in need of research. Subsequent advances indicate that ROS homeostasis is essential for ethylene regulation of plant growth and stress response (Steffens, 2014; Zhong et al., 2014; Yang et al., 2017). ROS is a double-edged sword during salt stress response. On the one hand, ROS act as important signal molecules to activate downstream metabolic pathways. Previous studies demonstrate that ROS burst via RESPIRATORY BURST OXIDASE HOMOLOG D (RbohD) and RbohF is essential for the Na^+^/K^+^ homeostasis in *Arabidopsis* (Ma et al., 2012), and ethylene-induced ROS production through transcriptional regulation on *AtRbohF* confers enhanced salt tolerance to the ethylene overproduced mutant *eto1* (Jiang et al., 2013). On the other hand, ethylene signaling component EIN3/EIL1 activates ROS-scavenging gene expression to deter excess ROS accumulation and to increase salt tolerance (Peng et al., 2014). Similarly, the effects of ethylene signaling downstream factors on ROS are inconsistent during different stages of various stresses. For example, ERF74 promotes ROS burst in the early stages of various stresses through the regulation of gene expression of *RbohD*, followed with induction of ROS-scavenging-related genes (Yao et al., 2017). However, ethylene inducible factor TERF1 improves stress tolerance through reduced ROS content (Zhang H. et al., 2016). Therefore, fine-tuning of ethylene biosynthesis and signaling on ROS homeostasis are critical for salt tolerance.

**Table 1:** Genes involved in ethylene- and AsA-mediated ROS homeostasis in response to salt stress.

Genes	Plant species	Treated material	Concentrations of NaCl	Treatment time	Treatment method	Regulation on salt stress	ROS metabolism
*ETO1, RBOHF*	*Arabidopsis*	4-week-old soil-grown plants	350 mM	7 days	Watering with NaCl solutions	Positive	Generation
*EIN3/EIL1, SIED1, POD*	*Arabidopsis*	5-d-old seedlings on MS medium	200 mM	3 days	Transferring to MS medium with NaCl	Positive	Scavenging
*ESE1*	*Arabidopsis*	5-d-old seedlings on MS medium	100 mM	7 days	Transferring to MS medium with NaCl	Positive	–
*JERF3, SOD*	Tobacco	5-d-old seedlings on MS medium	150 mM	-	Transferring to MS medium with NaCl	Positive	Scavenging
*ERF98, VTC1*	*Arabidopsis*	5-d-old seedlings on MS medium	180 mM	5–7 days	Transferring to MS medium with NaCl	Positive	Scavenging
*CSN5B*	*Arabidopsis*	Germinated seeds on MS medium	100 mM	10 days	Transferring to MS medium with NaCl	Negative	Generation
*SIZF3*	Tomato	5-week-old soil-grown plants	50–125 mM	21 days	Watering with NaCl solutions	Positive	Scavenging
*OsEIL1/OsEIL2*	Rice	8/9-d-old seedlings in hydroponic culture solution	200 mM	5 days	Transferring to NaCl-containing culture solution	Negative	–
*SIT1, MPK3/6*	Rice	10-d-old seedlings in hydroponic culture solution	200–250 mM	4 days	Transferring to NaCl-containing culture solution	Negative	Generation


Additionally, our previous studies verified several downstream regulators of ethylene signaling in salt response and ROS homeostasis. For example, ETHYLENE AND SALT INDUCIBLE ERF GENE 1 (*ESE1)*, a direct target gene of EIN3, positively regulates salt tolerance and coordinates with *EIN3* to activate downstream salt-related gene expression in *Arabidopsis* (Zhang et al., 2011); and *JERF3*, an ethylene-induced gene, enhances salt tolerance via direct modulation on the gene expressions of *SUPEROXIDE DISMUTASE* (*SOD*) and *CARBONIC ANHYDRASE* (*CA*) in tomato to eliminate ROS, which also confers drought and osmotic stress tolerance to transgenic rice with heterologous expression of *JERF3* (Wu et al., 2008; Zhang et al., 2010; Table 1). Thus, identification of more ethylene signaling downstream regulators participating in ROS homeostasis under salt stress is necessary for elucidating the regulation of ethylene on ROS and salt response.

### The Scavenging Role of AsA on ROS Homeostasis Contributes to Salt Tolerance

AsA, also known as vitamin C, is a low molecular weight antioxidant, functioning as a component of non-enzymatic scavenging of ROS in plant growth and stress tolerance (Smirnoff, 2000; Conklin, 2004; Akram et al., 2017). It has been reported that AsA improves salt tolerance in various species, including rice, potato, tomato, and citrus (Shalata and Neumann, 2001; Hemavathi et al., 2010; Kostopoulou et al., 2015; Qin et al., 2016). The L-galactose pathway is the main pathway of AsA biosynthesis in plants, and most of the genes in this pathway have been identified (Bulley and Laing, 2016). Investigations also have elucidated the regulation via the L-galactose pathway of AsA biosynthesis, including the modulations on the AsA biosynthesis enzyme activities and stabilities at transcriptional and translational levels (Laing et al., 2015). One of these regulators is calmodulins-like 10, which interacts with AsA biosynthesis enzyme phosphomannomutase (PMM) to modulate enzyme activities and AsA pool (Cho et al., 2016), which suggested the role of calcium (Ca^2+^) in AsA biosynthesis. It has been known that Ca^2+^ signaling is triggered by ROS accumulation (Rentel and Knight, 2004) and Ca^2+^ wave is induced under salt stress (Choi et al., 2014; Liu et al., 2018). A chloroplast protein, QUASIMODO1 (QUA1), functions upstream of a thylakoid-localized Ca^2+^ sensor, CAS, to mediate Ca^2+^ signaling under salt stress (Zheng et al., 2017). Additionally, AsA could trigger increase of cytosolic Ca^2+^ in *Arabidopsis* as a signaling molecule (Makavitskaya et al., 2018), suggesting the association between Ca^2+^ sensor and AsA-mediated ROS scavenging during salt responses, and feedback regulation of Ca^2+^ signaling and ROS homeostasis.

The regulation factors of AsA biosynthesis also play a role in salt response. Our previous investigations have found that ethylene-induced factor AtERF98 enhances salt tolerance due to transcriptional activation on gene expressions of AsA biosynthesis enzymes, especially direct binding to the promoter of a key enzyme of AsA biosynthesis encoding gene *VTC1* (Zhang et al., 2012). Meanwhile, we also identified the post-transcriptional modulation of COP9 SIGNALOSOME SUBUNIT 5B (CSN5B) on VTC1 in *Arabidopsis* (Wang J. et al., 2013; Li et al., 2016), elucidating a mechanism of light/dark effects on AsA contents. Loss-of-function mutant *csn5b*, with more AsA content and less ROS pool, displays increased salt tolerance, suggesting the positive regulation of AsA on salt response. Recent studies showed that salt induced zinc-finger protein SIZF3, which interferes with the interaction between CSN5B and VTC1, simultaneously promotes AsA accumulation and enhances salt tolerance (Li et al., 2018; Table 1). Thus, increasing AsA content is a potential approach for improving plant salt tolerance.

### The Integration of Ethylene in AsA Production Finetunes ROS Homeostasis Under Salt Stress

As discussed above, both ethylene and AsA could enhance salt tolerance via regulation of ROS homeostasis. Previous reports have indicated that ethylene in many cases maintains a low level of ROS contents under salt stress through the enzymatic pathway (Wu et al., 2008; Peng et al., 2014; Zhang W. et al., 2016). Moreover, the non-enzymatic pathway of scavenging ROS also participates in ethylene-mediated salt response, such as AtERF98, suggesting that the modulation of ethylene on ROS elimination is alternatively dependent on non-enzymatic antioxidant (Zhang et al., 2012).

**FIGURE 1:**
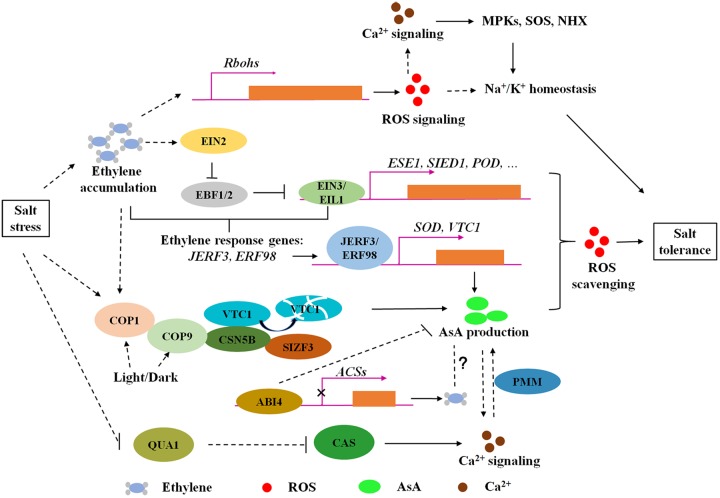
The modulation of ethylene signaling and AsA biosynthesis regulators on ROS homeostasis in response to salt stress. Ethylene is accumulated and plays dual roles in ROS homeostasis under salt stress. On one hand, ethylene promotes ROS production to active Na^+^ and K^+^ transport through upregulating *Rbohs* gene expression. In the other hand, salt stress enhances EBF1/EBF2 degradation through EIN2C-dependant translational regulation to increase EIN3/EIL1 protein levels, activating gene expression of EIN3 direct binding targets (*ESE1, SIED1*, and *POD*) and ethylene response factors (*JERF3* and *ERF98*) to regulate salt tolerance via ROS scavenging. ERF98 positively regulates salt tolerance via transcriptional activation of AsA biosynthesis gene *VTC1*. Moreover, CSN5B, a subunit of photomorphogenic COP9 signalosome, contributes to AsA biosynthesis and salt responses due to modulation on VTC1 degradation. SIZF3 also confers salt tolerance through mediating the interaction between CSN5B and VTC1. This research indicates that ROS accumulation under salt stress could be eliminated through enzymatic and non-enzymatic pathways, in both of which ethylene signaling is involved. However, the understanding of ethylene roles in AsA biosynthesis is yet limited. ABI4 negatively regulates ethylene synthesis and AsA production, which supply a possible mechanism coordinating ABA and ethylene to regulate AsA biosynthesis under salt stress. Ca^2+^ signaling could be induced by both ROS signaling and participates in AsA biosynthesis modulation through PMM. Arrows and lines with bars indicate activation and inhibition, respectively. Dotted lines indicate indirect regulations.

There are many environmental factors affecting AsA biosynthesis, such as light (Fukunaga et al., 2010), circadian rhythm (Dowdle et al., 2007), and high temperature (Richardson, 2004). CSN5B, identified in our previous studies (Wang J. et al., 2013), is a subunit of photomorphogenic COP9 signalosome (Gusmaroli et al., 2004), which acts together with COP1, COP10, and DET1 to repress photomorphogenesis (Yanagawa et al., 2004). This research suggest that CSN5B-regulated AsA biosynthesis is a part of photomorphogenesis. Intriguingly, ethylene has functions in COP1 nucleocytoplasmic partitioning (Yu et al., 2013, 2016), indicating a possible link between ethylene and light-regulated AsA biosynthesis. It was reported that ABA-INSENSITIVE 4 (ABI4) mediates AsA-regulated plant growth (Kerchev et al., 2011) and ethylene production via transcriptional repression of *ACS* in *Arabidopsis* (Dong et al., 2016). In this regard, ethylene seems to have crosstalk with ABA to modulate AsA production (Figure 1). However, the mechanisms for these modulations are yet to be elucidated.

## Conclusion and Perspectives

Emerging evidence provides the understanding of the roles of ethylene and AsA in salt tolerance through fine-tuning ROS homeostasis. Ethylene biosynthesis could be induced under salt stress, followed with ROS accumulation through transcriptional activation of *Rbohs* gene expression, in which ROS functions as a signal to regulate Na^+^/K^+^ homeostasis. Excessive ROS is toxic to plants, and ethylene also performs a scavenging role on ROS homeostasis under salt stress through signaling pathways, including the stability of EBF1/EBF2 and transcriptional regulation of EIN3/EIL1 on downstream direct or indirect regulators such as *ESE1*, *SIED1*, *POD*, *JERF3*, and *ERF98*. Ca^2+^ signaling, as a second messenger, could be induced by both ROS and AsA-mediated ROS balance, and participates in AsA biosynthesis modulation (Figure 1). The antagonistic effect of ethylene on ROS synthesis and scavenging under salinity stress is due to different functions of ROS at different developmental stages and in different tissues (Jiang et al., 2013; Peng et al., 2014). Additionally, non-enzymatic antioxidant AsA and the modulators involved in AsA biosynthesis confer to salt tolerance through reduced ROS accumulation (Figure 1; Wang J. et al., 2013; Qin et al., 2016; Li et al., 2018). However, the individual or crosstalk of ethylene and AsA regulation mechanisms on salt responses remain in need of further research. For example, although the components of the ethylene signaling pathway are conserved in *Arabidopsis* and rice (Yin et al., 2017), the underlying mechanisms of ethylene signaling in response to salt stress are different. Similarly, the effect of ethylene on plant growth is opposite in light and dark, such as hypocotyl elongation (Yu and Huang, 2017). Moreover, emerging research demonstrates that light plays a pivotal role in AsA synthesis (Fukunaga et al., 2010; Wang J. et al., 2013). These findings suggest a complex network regulated by ethylene signaling under different growth conditions. Further engagement is needed to determine whether ethylene and light coordinate AsA production to maintain ROS homeostasis during salt response. Furthermore, it is widely recognized that ABA and ethylene are simultaneously involved in stress responses (Kumar et al., 2016). ABA signaling component ABI4 mediates AsA-regulated plant growth (Kerchev et al., 2011) and inhibits ethylene biosynthesis (Dong et al., 2016; Figure 1). Nevertheless, the crosstalk between ethylene and ABA in the control AsA pool is unclear. Proper redox homeostasis is necessary for plant growth under salt stress; thus, making clear the detailed mechanisms of ethylene and AsA in maintaining ROS homeostasis will provide new insights for salt-tolerant genetic improvement.

## Author Contributions

RH proposed the concept. JW organized and drafted the manuscript. RH contributed to the editing of the manuscript. Both authors read and approved the manuscript.

## Conflict of Interest Statement

The authors declare that the research was conducted in the absence of any commercial or financial relationships that could be construed as a potential conflict of interest.
